# Factors of influence on acute skin toxicity of breast cancer patients treated with standard three-dimensional conformal radiotherapy (3D-CRT) after breast conserving surgery (BCS)

**DOI:** 10.1186/1748-717X-7-217

**Published:** 2012-12-18

**Authors:** Uta Kraus-Tiefenbacher, Andreas Sfintizky, Grit Welzel, Anna Simeonova, Elena Sperk, Kerstin Siebenlist, Sabine Mai, Frederik Wenz

**Affiliations:** 1 Department of Radiation Oncology, University Medical Center Mannheim, University of Heidelberg, 68167, Mannheim, Germany

**Keywords:** Skin toxicity, Breast cancer, External beam radiotherapy

## Abstract

**Purpose/Objectives:**

Standard 3D-CRT after BCS may cause skin toxicity with a wide range of intensity including acute effects like erythema or late effects. In order to reduce these side effects it is mandatory to identify potential factors of influence in breast cancer patients undergoing standard three-dimensional conformal radiation therapy (3D-CRT) of the breast and modern systemic therapy.

**Materials/Methods:**

Between 2006 and 2010 a total of 211 breast cancer patients (median age 52,4 years, range 24–77) after BCS consecutively treated in our institution with 3D-CRT (50 Gy whole breast photon radiotherapy followed by 16 Gy electron boost to the tumorbed) were evaluated with special focus on documented skin toxicity at the end of the 50 Gy-course. Standardized photodocumentation of the treated breast was done in each patient lying on the linac table with arms elevated. Skin toxicity was documented according to the common toxicity criteria (CTC)-score. Potential influencing factors were classified in three groups: patient-specific (smoking, age, breast size, body mass index = BMI, allergies), tumor-specific (tumorsize) and treatment-specific factors (antihormonal therapy with tamoxifen or aromatase inhibitors, chemotherapy). Uni- and multivariate statistical analyses were done using IBM SPSS version 19.

**Results:**

After 50 Gy 3D-CRT to the whole breast 28.9% of all 211 patients had no erythema, 62.2% showed erythema grade 1 (G1) and 8.5% erythema grade 2. None of the patients had grade 3/4 (G3/4) erythema.

In univariate analyses a significant influence or trend on the development of acute skin toxicities (erythema G0 versus G1 versus G2) was observed for larger breast volumes (p=0,004), smoking during radiation therapy (p=0,064) and absence of allergies (p=0,014) as well as larger tumorsize (p=0,009) and antihormonal therapy (p=0.005).

Neither patient age, BMI nor choice of chemotherapy showed any significant effect on higher grade toxicity. In the multivariate analysis, factors associated with higher grade skin toxicity were larger breast target volume (p=0,003), smoking (p=0,034) and absence of allergies (p=0,002).

**Conclusion:**

Patients treated in this study showed less objectively documented skin toxicity after 50 Gy 3D-CRT compared to similar patient cohorts. Factors associated with higher grade skin toxicity were smoking during 3D-CRT, absence of allergies and larger breast volumes.

## Introduction

Adjuvant external beam radiotherapy (EBRT) to the breast is standard of care after breast-conserving surgery (BCS) for patients with early-stage breast cancer, in order to achieve equivalent survival rates compared to those patients treated with mastectomy [1].

Although potential side effects can be reduced by using modern treatment techniques and fractionation schedules, skin erythema, breast edema and breast fibrosis are the most common side effects after EBRT. Skin toxicity is the predominant acute side effect of radiotherapy to the breast, occuring during or after EBRT in more than 90% of all patients. 10% of these patients develop CTC (common toxicity criteria version 2.0) G3 3 erythemas [2,3] that may limit the patients quality of life and compliance. The severity of skin reaction varies from mild erythema to moist desquamation and occasionally ulceration of the skin. Radiotherapy treatment related factors such as fractionation dose, beam energy and treatment technique can influence the severity of skin toxicity as can patient related factors such as breast size, breast geometry and smoking: Patients with larger breast volumes developed more severe skin toxicity during radiotherapy compared to patients with smaller breast volumes and wound-healing of women, who were smoking during EBRT, was delayed, compared to that of non-smokers [4]. This present study was designed to assess influencing factors during EBRT of the breast in a patient cohort treated in our institution. All patients included in this study were treated with 3 –dimensional, CT (computer tomography) - planned conformal radiotherapy (3D-CRT) and, according to receptor status and individual risk, modern systemic therapy.

## Materials and methods

211 breast cancer patients (median age 52,4 years, range 24–77) with histologically documented diagnosis of breast cancer were eligible for this study. Patients were treated at the department of Radiation Oncology, University Medical Center Mannheim, University of Heidelberg, Germany with standard 3D-CRT of the breast after breast conserving surgery. A three-dimensional treatment plan (OTP= Oncentra Treatment Plan® Version 3.3 (Nucletron BV, Veenendaal, Netherlands) was calculated individually for each patient. The breast target volume of each patient was measured via OTP treatment planning system by contouring manually each slice of breast tissue on planning CT. The treatment schedule comprised whole breast photon radiotherapy with energies ranging between 6 and 23 MV (see Table 1) up to a total dose of 50 Gy followed by 16 Gy electron boost to the tumorbed. Two Electa (Electa Precise Treatment System™) linear accelerators were used. Daily fractionation was 2.0 Gy. 3D-CRT was performed according to the International Commission on Radiation Units and Measurements (ICRU 50) criteria. In order to minimize or reduce possible skin irritation,general intructions for skin care during 3D-CRT were given to all patients before starting radiotherapy: patients should use mild skin (“baby”) powder once or twice a day during 3D-CRT and thereafter until skin recovered to normal.and treat themselves with local standard of care for the irradiated breast consisting of deodorant-free After 50 Gy 3D-CRT to the entire breast a standardized (arms elevated lying at the linear accelerator table) digital photograph of the irradiated breast and the intended boost field was done for each patient and saved in the electronic patient chart system Mosaiq (Radiation Oncology Information System, Electa AB, Stockholm, Sweden). All photos were made with the same camera, the same technique and identical lighting inside the radiation room.

**Table 1:** Summary of all relevant patient characteristics: patient -, tumour- and treatment related parameters

**Patient characteristics (at the beginning of EBRT)**	**Median**	**Range**
**Age** [years]	52.4	24.3-77.3
**Patient weight** [kg]	65	44-120
**Patient height** [cm]	166	150-180
**Body-Mass-Index**	23.9	17.4-41.8
**Breast target volume** [cc]	946.9	402-4283
**Smoking**	**n**	**%**
· no current smoking	146	69.2
· current smoking	41	19.4
· unknown	24	11.4
**Allergies**		
· with allergies	71	33.6
· without allergies	140	66.4
**Tumour characteristics**	**n**	**%**
**Tumour localization**		
· right-sided	100	47.4
· left-sided	110	52.1
· both sides	1	0.5
**Histology**		
· ductal-invasive	137	64.9
· ductal-invasive + DCIS	29	13.7
· lobular-invasive	15	7.1
· mixed (ductal- and lobular-invasive)	15	7.1
· other	15	7.1
**T-stage**		
· 1	143	67.8
· 2	64	30.3
· 3	3	1.4
· 4	1	0.5
**Treatment characteristics 3D-CRT**	**Median**	**Range**
**Interval BCS – start of 3D-CRT** [days]	74	19-393
**Interval start of 3D-CRT-boost** [days]	38	30-79
**Energy levels**	**n**	**%**
· 6 MV	168	79.6
· 6 MV/18 MV	39	18.5
· 6 MV/18 MV/23 MV	4	1.9
**Systemic therapy**		
**Chemotherapy**		
· no chemotherapy	104	49.3
· Anthracyclines	59	28.0
· Taxanes	1	0.5
· Anthracyclines and taxanes	45	21.3
· neither anthracyclines nor taxanes	2	1.0
**Antihormonal therapy**		
· no antihormonal therapy	51	24.2
· Tamoxifen	111	52.6
· Aromatase inhibitors	49	23.2
**Herceptin**		
· no	191	90.5
· yes	20	9.5

All data, including medical history including history of allergies, diagnosis, pathology and treatment reports were retrospectively evaluated from Mosaiq with special focus on documented skin toxicity (Common toxicity criteria CTC version 2.0: grade 0 =°0, grade 1 =°I, grade 2 =°II, grade 3 =°III) at the end of the 50 Gy -course. For that reason all photodocumentions were evaluated independently by two radiation oncologists blinded for the clinical data. Uni- and multivariate statistical analyses were done using IBM SPSS Version 19. For statistical analyses, as appropriate, chi-square test (or Fisher's exact test), t-test for independent samples (or Mann–Whitney U-test for independent samples), one way ANOVA (or Kruskal Wallis ANOVA on ranks) and multiple ordinal regression analysis was used. A significance level of 5% was chosen.

Skin toxicity was scored according to the common toxicity criteria (CTC version 2.0)-score. Potential influencing factors were classified in three groups in order to keep track on all parameters patient-specific (smoking, age, breast size, BMI, allergies), tumor-specific (tumorsize) and treatment-specific factors (antihormonal therapy with tamoxifen or aromatase inhibitors, chemotherapy).

Table 1 gives a full summary of all relevant patient characteristics according to patient-, tumour- and treatment characteristics.

## Results

211 breast cancer patients treated between 2006 and 2010 at the department of Radiation Oncology, Mannheim Medical Centre, University of Heidelberg, Germany for adjuvant EBRT were enrolled to this study. Median interval between BCS and beginning of EBRT was 74 days (19–393), median interval between beginning of EBRT and the 50 Gy time point was 38 days (30–79). After 50 Gy EBRT to the whole breast 28.9% of all patients had no erythema, 62.6% showed an erythema G 1 and 8.5% an erythema G 2. None of the patients had °III/IV erythema.

In univariate analyses larger tumour size (p=0.009), antihormonal therapy with aromatase inhibitors (p=0.005), larger breast volumes (p=0.004), smoking during EBRT (p=0.064) and absence of allergies (p=0.014) had an influence on skin toxicity after 3D-CRT (i.e. erythema G0 versus G1 versus G2).

### Breast volume

**Figure 1:**
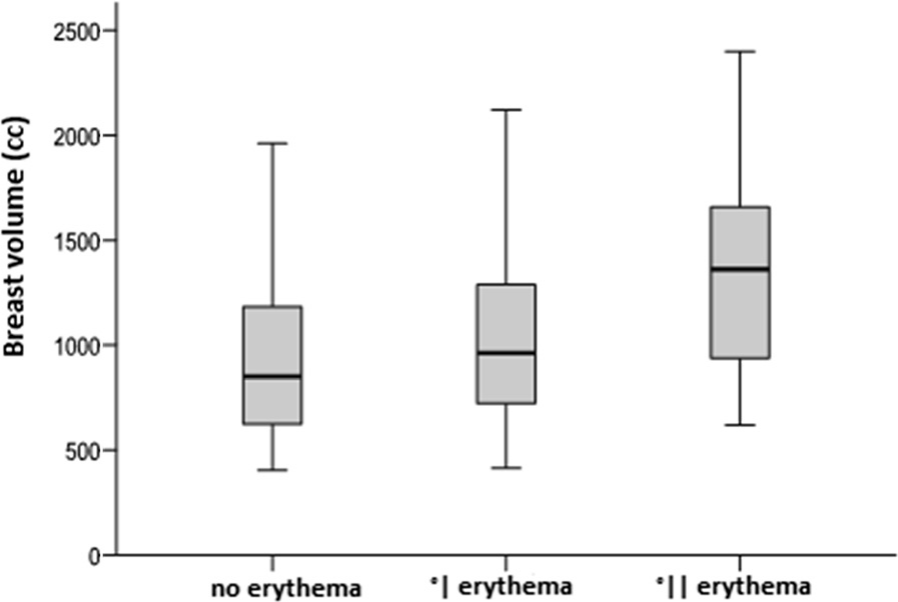
Breast volume and erythema after 50 Gy 3D-CRT (p= 0,009).

Figure 1: Breast volume and erythema after 50 Gy 3D-CRT (p= 0,009) .

### Smoking

**Figure 2:**
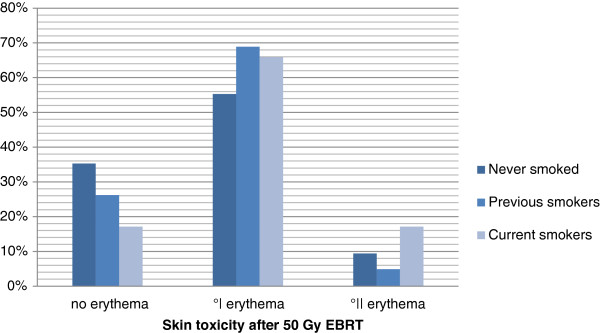
Smoking and skin toxicity after 50 Gy 3D-CRT (p= 0,064).

Figure 2: Smoking and skin toxicity after 50 Gy 3D-CRT (p= 0,064) .

#### Allergies

From all 71 patients with allergies 28 (39%) showed no skin toxicities at all, 41 (58%) had erythema G1 and 2 (3%) erythema G2. From patients without allergies 33 (24%) showed no skin toxicities at all, 91 (65%) had erythema G1 and 16 (11%) erythema G2.

**Figure 3:**
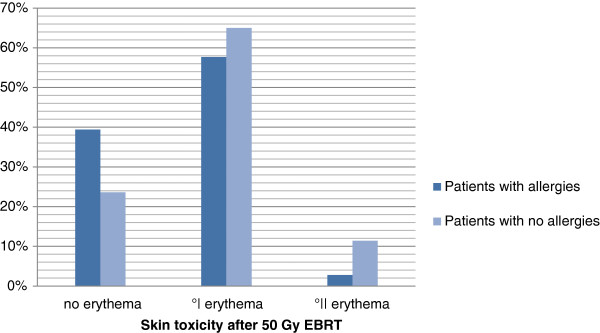
Allergies and skin toxicity after 50 Gy 3D-CRT (p=0.014).

Figure 3: Allergies and skin toxicity after 50 Gy3D-CRT (p=0.014) .

#### T-stage

**Figure 4:**
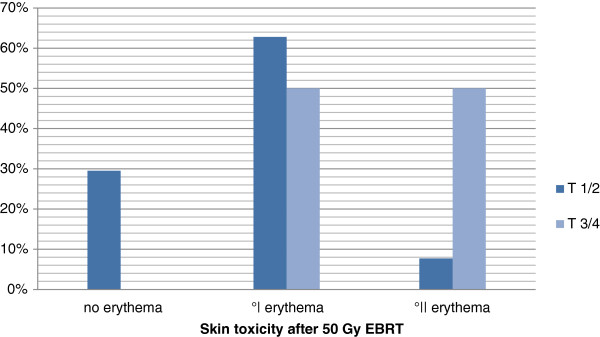
T-stage and skin toxicity after 50 Gy 3D-CRT.

The distribution of patients with no erythema, erythema G1 and erythema G2 is shown in Table 2 and Figure 4.

**Table 2:** T-stage and skin toxicity after 50 Gy 3D-CRT

**T-stage** **(n)**	**No erythema n (%)**	**Erythema G 1grade 1 n (%)**	**Erythema G 1grade 1I n (%)**
T1/2 ( 207)	61 (29.5)	130 (62.8)	16 (7.7)
T3/4 (4)	0 (0)	2 (50)	2 (50)
All patients (211)	61 (28.9)	132 (62.6)	18 (8.5)

#### Antihormonal therapy

Patients with aromatase inhibitors were subclassified according to their prescribed compound: 24 patients (11,4%) were treated with Anastrozole (Arimidex®), 24 (11,4%) with Letrozole (Femara®) and one patient (0,5%) with Exemestan (Aromasin®). Increased acute skin toxicity (G 1 erythema) for patients taking Anastrozole followed by Letrozole was found (p=0,01).

Table 3: Choice of antihormonal therapy and skin toxicity after 50 Gy 3D-CRT (p=0.005) .

**Table 3:** Choice of antihormonal therapy and skin toxicity after 50 Gy 3D-CRT (p=0.005)

		**No erythema**	**Erythema G1**	**Erythema G2**
No antihormonal therapy	n	13	35	3
	%	25.5	68.6	5.9
Tamoxifen	n	28	77	6
	%	25.2	69.4	5.4
Anastrozole	n	9	9	6
	%	37.5	37.5	25
Letrozole	n	10	11	3
	%	41.7	45.8	12.5
Exemestan	n	1	0	0
	%	100	.0	.0
Total	n	61	132	18
	%	28.9	62.6	8.5

To summarize, patients with aromatase inhibitors (n=49) developed more often erythema G2 compared to patients with no antihormonal therapy or with tamoxifen. This difference was significant (p=0.005).

**Figure 5:**
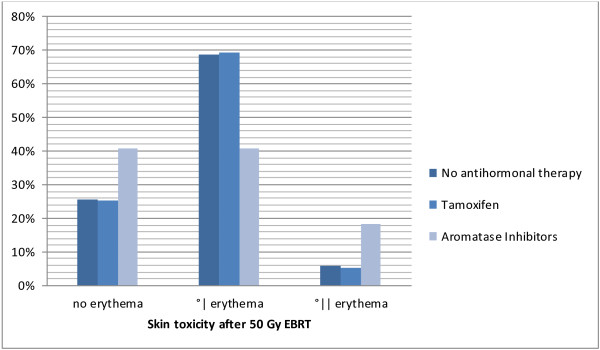
Antihormonal therapy and skin toxicity after 50 Gy 3D-CRT (p=0.005).

Figure 5: Antihormonal therapy and skin toxicity after 50 Gy 3D-CRT (p=0.005) .

All other parameters such as age, BMI, and chemotherapy had no influence on the development of increased skin toxicities.

Table 4: Age, BMI, Chemotherapy and skin toxicity after 50 Gy EBRT .

**Table 4:** Age, BMI, Chemotherapy and skin toxicity after 50 Gy EBRT

		** Median**	**Range**
**Age**	All	52.42	24.25-77.29
	No erythema	51.87	29.43-77.29
	Erythema °I	52.25	24.25-73.76
	Erythema °II	58.05	32.38-68.43
**BMI**	All	23.9	17.4-41.79
	No erythema	23.31	17.93-40.56
	Erythema °I	24.0	17.4-41.79
	Erythema °II	25.51	17.53-39.79
		** n**	**%**
**Chemotherapy**	No erythema	27	26
	Erythema °I	67	64.4
	Erythema °II	10	9.6
**No chemotherapy**	No erythema	34	36.4
	Erythema °I	65	69.6
	Erythema °II	8	7.5

The results of multivariate analysis are summarized in Table 5. There were significant influences of larger breast target volume (p=0.003), smoking during EBRT (p=0.034) and absence of allergies (p=0.002). Neither patient age, BMI nor adjuvant chemotherapy nor type of chemotherapy (taxanes versus anthracyclines) showed a significant effect on higher grade acute skin toxicity.

**Table 5:** Results of the multivariate analysis (ordinal regression analysis) erythema G 0 versus erythema G 1 versus erythema G 2 at 50 Gy time point

	**Estimate**	**SE**	**Wald**	**df**	**p**	**95% CI**
Breast volume [cc] (continuous)	.001	.000	9.031	1	.003	.000; .002
No allergies vs. allergies	1.013	.334	9.191	1	.002	.358; 1.669
T1/2 vs T2/4	1.482	1.090	1.851	1	.174	−3.618; .653
AHT aromatase inhibitors vs No AHT	.019	.376	.003	1	.959	-.718; .757
AHT aromatase inhibitors vs No AHT	-.265	.455	.341	1	.559	−1.156; .625
No current Smoking vs current smoking	-.819	.386	4.498	1	.034	−1.575; .062

## Discussion

Progress has been made during the last few years in order to reduce the side effects of EBRT after breast-conserving surgery: immobilization devices and modern radiation techniques like 3D-conformal radiotherapy or intensity-modulated radiotherapy (IMRT) [5,6]. Although using modern and highly complex techniques with high energy photons, going along with decreased skin dose and improved dose-homogeneity, there will always occur skin toxicity during or after radiation therapy [7,8]. Literature reports erythema rates after standard 3D-CRT at an average of 90% of the patients, while 10% developed erythema G 3 or 4 10 years ago [2]. However breast cancer treatment includes nowadays other chemotherapies, other antihormonal therapies and improved radiation therapy techniques. Patients from our study show lower skin toxicity compared to the data from the literature: we found only 70% erythema G 1 or 2 and no G3 erythema at all. However the 211 patients from our study were evaluated after a total dose of 50 Gy, with a single fractionation of 2.0 Gy, while some other authors have collected their data at a higher dose level or later point of time [7].

The individual reaction of the skin to EBRT with high-energy photons is complex and depends on numerous factors that can be patient-, tumour- and/or treatment-related.

Previous studies have tried to identify potential factors associated with increased radiotherapy –induced toxicity [8], but many of these studies found no association between skin toxicity and the above mentioned parameters at all [9,10]. However the number of included patients was often too low to find any significant results [11,12]. Furthermore the scoring of the individual skin toxicity is quite subjective and inaccurate. Observing and evaluating the photodocumentations at a clearly defined time point in each of our 211 patients by two independent observers, we tried to get a more objective rating of individual skin toxicity. We believe that our standardized photodocumentation in each patient at a defined time point and scoring according to CTC-score makes our results objective and useful, while other studies often collect their results at different time points using more subjective criteria.

Regarding all parameters of our study with potential influence on skin toxicity, we found significant correlations in uni- as well as in multivariate analyses only for the three patient-related factors breast volume, current smoking and absence of allergies.

### Higher breast volumes

Patients with larger breast volumes developed in our study significantly more erythema G 1 and 2 compared to patients with smaller breast volumes.

A study from Vicini and colleagues could show comparable results: patients with breast volumes > 1600 cc had more acute skin toxicity compared to those with breast volumes < 1000 cc [13]. Another study showed 27% RTOG G 2 erythema and 0% G 3 erythema in patients with breast volumes < 975 cc, while patients with breast volumes > 1600 cc developed 59% RTOG G 2 and 3% G 3I erythema (p=0,002) [5]. Breast volumes were measured in both studies in analogy to our method (manually contouring of breast target volume).

In another study with a positive correlation between breast size and skin toxicity published in 2006 larger breast volumes (here defined as > cup D) were significantly associated with higher graded (> G2) skin toxicity [14], however graduating breast volumes in different cup sizes seem to us to remain more inaccurate compared to 3D volumetry.

Fisher and colleagues summarized their data concerning breast volume and skin toxicity as follows: patients with small breasts developed ≥ erythema G1 in 11–21%, patients with medium sized breasts in 36-39% and patients with large breasts in 43-50% [4].

A possible explanation for this correlation might be that EBRT treatment plans of larger breast volumes have much more dose inhomogeneities than that of smaller breasts. These dose inhomogeneities may lead to partial hot spots followed by increased skin toxicities and can better be balanced with IMRT techniques [5,15] especially for patients with larger breast sizes.

### Current smoking

All 211 patients were classified in two groups: no current versus current smoking, Our observation that patients, who were smoking during EBRT develop significantly more skin toxicity, goes along with a study from Wells et al. with 357 patients randomized to apply aqueous cream, sucralfate cream or no cream, who published increasing skin reactions during EBRT in each of the three groups in correlation to no smoking, previous smoking and current smoking patients [16]. Another group also confirmed smoking as predictive factor for increased skin toxicity [17] in breast cancer patients. There are also results from other tumour entities, who describe a positive correlation between smoking during radiation therapy and increased skin toxicity evaluating, for example, patients with anal cancer [18].

### Allergies

The development of skin erythemas during radiation therapy is accompanied by perivascular inflammation going along amongst others with an accumulation of mastocytes and cytokines. Due to the fact that patients with allergies show permanently more mastocytes in their skin, we had expected that this patient group would develop more skin toxicity after EBRT. There are a few studies dealing with manipulation of this inflammatory reaction during EBRT by topical agents with steroids in order to reduce the skin reactions during radiation therapy. The results of three studies with restricted numbers of patients showed concordantly that patients treated with steroid ointments during EBRT developed significantly less moist skin reactions [17,19,20]. Porock, in contrast, could not show any benefit of steroid products used during radiotherapy [21].

In our study patients with the history of allergies developed in both uni- and multivariate analyses significantly less erythemas compared to patients with no allergies. But a clear explanation for this result besides the statistical significance is still missing.

### T-stage

Some authors describe a positive correlation between larger resected tissue volume and higher grade skin toxicity during or after EBRT [7,22], however it is apparently difficult to exactly differentiate between bad cosmetic outcome and increased skin toxicity. There are only 4 patients from our cohort with T3/4-stage disease developing half and half erythema G1 and 2, therefore it is impossible to draw any conclusion regarding this point.

### Anthormonal therapy

A quarter of our patient group received no antihormonal therapy, about half antihormonal therapy with tamoxifen and another quarter upfront therapy with aromatase inhibitors.

There was no significant difference between skin toxicity and in patients taking no antihormonal therapy or taking tamoxifen, but between taking no antihormonal therapy and taking aromatase inhibitors. Patients with aromatase inhibitors showed significantly more grade II erythemas compared to the other two groups.

Numerous studies confirm the finding that there was no difference between skin toxicity whether taking no antihormonal medication or taking tamoxifen [23-25]. Our observation that patients with aromatase inhibitors develop more skin toxicity after EBRT than patients without antihormonal therapy or tamoxifen does not coincide with that described in literature [26,27], where concordantly no disadvantage with regard to aromatase inhibitors and EBRT could be found. The subclassification of different aromatase inhibitors is of restricted value due to small groups.

In contrast to the results of several studies with positive correlation between body mass index (BMI) and skin toxicity [6,15,22,23], for our patients BMI had no positive predictive value, perhaps due to the fact that the BMI for our total patient cohort was only 23.9 (median) and for this reason ranging below the threshold of 25 [28,29] with a higher risk of skin toxicity.

Whether chemotherapy before EBRT has a negative impact on skin toxicity or not, remains unclear. In addition to our data, there are a few studies that did not find a significant correlation between chemotherapy and increased skin toxicity [5,13], on the other hand other published analyses showed a trend to increased higher grade skin toxicities after chemotherapy [7,22], at which patients treated with anthracyclines seem to develop more skin reactions after EBRT [30].

## Conclusion

Our retrospective analysis showed less acute skin toxicity to comparable patient cohorts treated 10 years ago. Only the patient specific factors larger breast volumes, smoking during EBRT and absence of allergies showed significant influence in multivariate analyses, whereas larger BMI or receiving chemotherapy before radiotherapy did not correlate with higher skin toxicity. Larger T-stages and treatment with aromatase inhibitors showed a positive trend on the development of higher grade erythema. Consequently patients should be advised not to smoke. Individualized radiation treatment with IMRT is justified or even recommended in patients with larger breast volumes (> 1600 cc).

## Competing interests

The authors declare no conflict of interest.

## Authors’ contribution

UKT drafted the manuscript, AS collected the data and evaluated all patient photographs, GW was responsible for the statistical work , AS collected the data and evaluated all patient photographs, ES, KS and SM participated in treatment planning, patient treatment and follow-up, FW developed the concept and drafted the manuscript. All authors read and approved the final manuscript.
